# A Brief Online Intervention Based on Dialectical Behavior Therapy for a Reduction in Binge-Eating Symptoms and Eating Pathology

**DOI:** 10.3390/nu16162696

**Published:** 2024-08-14

**Authors:** Silvia Cerolini, Monica D’Amico, Andrea Zagaria, Edoardo Mocini, Generosa Monda, Lorenzo Maria Donini, Caterina Lombardo

**Affiliations:** 1 Department of Psychology, Sapienza University of Rome, 00185 Roma, Italy or s.cerolini@unimarconi.it (S.C.); monica.damico@uniroma1.it (M.D.); andrea.zagaria@uniroma1.it (A.Z.); generosa.monda@gmail.com (G.M.); 2 Department of Human Sciences, Guglielmo Marconi University, 00193 Roma, Italy; 3 Department of Experimental Medicine, Sapienza University of Rome, 00185 Roma, Italy or edoardo.mocini@uniecampus.it (E.M.); lorenzomaria.donini@uniroma1.it (L.M.D.); 4 Department of Theoretical and Applied Sciences, eCampus University, 22060 Novedrate, Italy

**Keywords:** online intervention, group intervention, binge-eating disorder, disordered eating, bulimia, dialectical behavior therapy, DBT

## Abstract

Dysregulated eating behaviors, comprising subthreshold and clinical binge-eating disorder (BED) and bulimia nervosa (BN), are increasing among the general population, with a consequent negative impact on one’s health and well-being. Despite the severity of these outcomes, people with BED and BN often face a delay in receiving a diagnosis or treatment, often due to difficulties in accessing care. Hence, evidence-based and sustainable interventions for eating symptomatology are needed. The present study aims to assess the effectiveness of a web-based 10-session multidisciplinary group intervention based on Dialectical Behavior Therapy (DBT) for BED and BN, aimed at reducing psychological distress and binge-eating-related symptomatology in a sample of patients with dysregulated eating behaviors and including one session of nutritional therapeutic education. A total of 65 participants (84.6% F; age M = 38.5 ± 13.2; experimental group, N = 43; treatment-as-usual group, TAU, N = 22) took part in the study. The results show, after the 9 weekly sessions, a significant reduction in binge-eating-related symptomatology and general psychopathology and an increased self-esteem and eating self-efficacy in social contexts in the experimental group compared to the treatment-as-usual group (T0 vs. T1). Improvements in the experimental group were significantly maintained after one month from the end of the intervention (T2) in terms of binge-eating symptoms, general psychopathology, and eating self-efficacy in social contexts. This study supports the effectiveness of a brief web-based multidisciplinary group intervention in reducing eating symptomatology and psychological distress and enhancing self-esteem and eating self-efficacy in a group of people with dysregulated eating behaviors. Brief web-based interventions could represent an accessible and sustainable resource to address binge-eating-related symptomatology in public clinical settings.

## 1. Introduction

Eating disorders (EDs) are characterized by dysfunctional eating behaviors that could be accompanied by an altered state of nutrition (i.e., malnutrition, undernutrition, or overnutrition) [1]. EDs are widely prevalent in Western countries [2,3] and negatively impact one’s quality of life, resulting in impairment in different areas of functioning, such as one’s participation and productivity in occupational and educational activities and social contexts and one’s emotional and psychological well-being [4]. In the last few decades, the prevalence of eating disorders has been rising abruptly, especially among women (from 3.5% in the period of 2000–2006 to 7.8% for the period of 2013–2018) [5]. Moreover, most EDs (i.e., bulimia nervosa, anorexia nervosa, and binge-eating disorder) are characterized by high rates of dropout from therapy [6,7] and relapse [8], and it is estimated that only one-third of patients are formally diagnosed [2]. When binge-eating symptoms are present, eating disorders are often associated with obesity [9,10]. Specifically, individuals with binge-eating disorder (BED) are 3–6 times more probable of living with obesity than those without an ED; moreover, research reports an earlier overweight onset and a history of childhood obesity [11]. Obesity and eating disorders often share risk factors, behavioral characteristics, consequences on one’s quality of life, and trends over time [12]. Obesity has been declared a global epidemic by the World Health Organization (WHO), as it increased from 30.5% in 1999–2000 to 42.4% in 2017–2018, constituting an all-cause risk of mortality [13]. In Italy, it was estimated that between 2016 and 2021, about 32% of the general population between 18 and 69 years old was overweight, and about 10.5% are in a condition of obesity [14]. Nevertheless, people with EDs and obesity, whether the conditions are co-existing or alone, often face a delay in receiving a diagnosis or treatment [15,16]. After the COVID-19 pandemic, the use of telehealth medicine increased significantly [17]. On the other hand, in-presence access to healthcare systems has become even more difficult for various reasons [16]. In fact, since the lockdown due to the pandemic, reduced social support, a disruption of daily routines, increased psychological distress, a fear of contagion, and worsened economic conditions were associated with decreased access to care [18]. The worsening of eating-disorder symptomatology during the pandemic [19,20,21,22,23,24] and the augmented difficulties in accessing public services [25,26] maintained after the emergency phase raised the need for more accessible and sustainable interventions [27]. Online interventions have demonstrated greater accessibility and affordability compared to face-to-face interventions [28]. They are increasingly showing efficacy for several psychological disorders, for example, depression, bipolar disorders, alcohol-use disorder, and EDs [29,30,31,32,33,34,35]. Specifically, web-based treatments for full or subthreshold BED have been shown to be effective, even though the evidence is still limited (i.e., only three Randomized Control Trials—RCTs—as reported by a current review and meta-analysis [36]). The interventions described in the studies included in the cited reviews mostly target BED and bulimia nervosa (BN) or their subthreshold respective disorders; moreover, they are mainly based on Cognitive Behavioral Therapies such as Internet-based CBT (I-CBT) or guided self-help (GSH). Recently, Högdahl et al. [37] compared two CBT online programs to an in-presence intensive group treatment in a clinical setting, finding that all three interventions were associated with decreased symptomatology both in post-intervention and a one-year follow-up. Melisse and colleagues [38] found that, in a sample of patients diagnosed with BED, guided self-help CBT-E improved binge-eating episodes, eating-disorder symptoms, and impairment due to the consequences of eating disorders, compared to a delayed-treatment control group. Taken together, these results support the potentiality of online interventions for eating disorders but also underline the necessity of more extensive evidence.

The present study aims to assess the effectiveness of a web-based brief and adapted version of a DBT-based intervention for full or subthreshold BED and BN. The intervention respects the current guidelines of the National Institute for Clinical Excellence (NICE) [39] and the American Psychiatric Association (APA) [40] for eating-disorder treatment [41], which recommends psychoeducation on the disorder, monitoring of one’s weight and mental and physical health; a multidisciplinary approach; and coordination between services where different professionals are in charge of the treatment: doctors, nutritionists/dieticians, psychologists, and psychiatrists.

## 2. Materials and Methods

### 2.1. The Study

This quasi-experimental study was conducted within the project “Determinants of quality of life in a sample of patients with altered nutritional status before and after integrated multidisciplinary nutritional rehabilitation treatment”, developed in cooperation between the Department of Experimental Medicine and the Department of Psychology at the Sapienza University of Rome and was approved by the Ethical Committee of “Policlinico Umberto I, Sapienza University of Rome” (Ref. 5915). This project aims to offer rehabilitation programs consistent with national [42] and international [39] guidelines.

The present study aims to assess the effectiveness of a web-based 10-session multidisciplinary group intervention in reducing psychological distress and binge-eating-related symptomatology in a sample of patients with dysregulated eating behaviors. The intervention was based on the DBT protocol for BED and BN [43,44] and included a therapeutical educational session on nutrition, dealing with disconfirming false myths about diet, food, and obesity management. All but the final educational sessions were conducted by trained psychologists. The educational sessions were conducted by a Physician Specialist in Nutritional Science.

### 2.2. Participants

Participants were recruited at the Integrated Service for Eating Disorders, comprising a network of services of the Sapienza University of Rome, including the Research Unit in Food and Nutrition Sciences (Department of Experimental Medicine), the Clinical Psychology and Counselling Service (Department of Psychology), and the Centre for High Specialization for the Treatment of Obesity (CASCO) of the Policlinico Umberto I–V Clinica Medica. Participants were enrolled between March 2020 and December 2023. Patients were assessed by a licensed psychologist through the Italian version of the Eating Disorder Examination, 17th edition (EDE 17.0D) [45,46], which is considered the gold standard for ED assessment and diagnostic evaluation [47]. The EDE 17.0D is a semi-structured interview focused on the eating symptomatology of the past 28 days, evaluating the presence of eating disorders in the past three months. Those who were diagnosed with BED or BN or who presented a disordered eating behavior (i.e., subthreshold BED or BN) according to the EDE 17.0D scores were selected. Exclusion criteria were the presence of severe psychopathology, as suggested by the authors of the original protocol [43], non-fluency in the Italian language, non-accessibility to a computer and/or a stable connection, and being younger than 18 years old.

A power analysis was conducted using G*Power version 3.1 in order to detect a within–between interaction of η_p_^2^ = 0.06 through a two-way mixed-design ANOVA, which is considered a medium effect size according to Cohen’s criteria. Assuming a power of 0.80 and a significance criterion of 0.05, the minimum sample size needed was 34.

### 2.3. Procedure

Participants deemed eligible, according to the medical and psychological evaluation described above, were invited to participate in the intervention. Participants who agreed to participate were allocated to the treatment group. Eligible people who were selected after the maximum number of members in each experimental group was reached were invited to participate in a treatment-as-usual (TAU) waiting-list group and completed the same questionnaires as the treatment group. Participants in the TAU group were invited to participate in the intervention after completing the follow-up questionnaires. Both groups received the TAU intervention, which consisted of multidisciplinary nutritional counselling, including individualized diet therapy and monthly sessions with a physician and a psychologist. All participants were invited to answer the questionnaires described in the “Instruments” section at the baseline and after two (corresponding to the end of the intervention in the experimental group) and three months (corresponding to the follow-up of the experimental group) after the baseline evaluation. Questionnaires included informed consent. Participants in the control group were invited to complete the survey in the same time intervals as the experimental group. They were also invited to participate in the following edition of the experimental intervention. Sixty-seven patients were considered eligible and were invited to participate. Two participants dropped out during the intervention and were excluded from the study. The final sample consists of 65 participants (experimental group, N = 43; TAU group, N = 22) reporting either subthreshold or clinically relevant binge-eating symptoms (84.6% F; age M = 38.5 ± 13.2).

### 2.4. Intervention

The intervention was developed based on the *Dialectical Behavior Therapy for Binge Eating and Bulimia* by Safer and colleagues [43]. DBT is a third-wave cognitive–behavioral treatment that was originally developed for patients at high suicidal risk and who are affected by borderline personality disorder [48,49]. The dialectical approach intends to promote a synthesis between acceptance and change through the development of dialectical skills [49]. The skills addressed by dialectical–behavior interventions are mindfulness, distress tolerance, interpersonal effectiveness, and emotional regulation. An extensive body of literature has provided evidence of efficacy for dialectical–behavior interventions, for example, in reducing and stabilizing a series of self-harm symptoms [50] and in increasing emotion regulation [51] in patients with borderline personality disorder. Moreover, a recent meta-analysis found improved emotion regulation, reduced depressive symptoms, decreased eating-disorder psychopathology, fewer objective binge episodes, and a lower BMI after DBT interventions for BED and BN compared to a control group [52]. The protocol for BED and BN, originally developed as a 20-session in-presence skill training, was adapted by three of the authors (S.C., G.M., and C.L.) for the purposes of the present study into a web-based 10-session multidisciplinary intervention. It consisted of 9 sessions with two trained Psychologists specializing in Psychotherapy and one session with a Physician Specialist in Nutritional Science. A total of 10 licensed psychologists specializing in psychotherapy conducted the various editions of the group training in couples. Conductors were trained and supervised by three psychotherapists experts in EDs, and participated in a previous edition as observers. Sessions were scheduled weekly and lasted 2 h. Meetings were held online via Google Meet. Groups included five to seven participants who could interact with each other during the online sessions. It was made sure that participants did not know each other previous to the intervention. The first part of the first meeting was dedicated to introducing the participants to each other and welcoming them in order to make them comfortable. The leading psychologists left each participant free to interact with each other, mediating between them only when necessary. All the details about the intervention (i.e., theoretical framework, content of each session, and structure) are available in the Supplementary Materials.

### 2.5. Instruments

The questionnaires were completed on the Qualtrics platform (www.qualtrics.com, accessed on 24 June 2024). Participants were invited to complete the survey at the baseline before the first meeting (T0), after the last meeting (T1), and one month after the end of the intervention (T2). Questionnaires included standardized instruments validated in Italian. Online completion of the survey took about 20–30 min. Patients did not report any negative effects related to the survey. No reimbursement or reward was provided for the intervention.

1. Demographic and health information. Name, surname, date of birth, age, educational qualification, profession, marital status, weight, and height. To calculate the Body Mass Index (BMI), the weight in kilograms was divided by the squared value of the height in meters (kg/m^2^).
2. Eating-behavior symptomatology. Patients completed the Eating Disorder Examination Questionnaire, 6th edition (EDEQ 6.0) [53], a 28-item questionnaire examining eating-behavior symptoms in the previous 28 days. Items 1 to 12 and 19 to 28 provide a response on scales of frequency or intensity from 0 to 6, ranging from “never” to “every day” or from “none” to “extreme”. These items are summed into four subscales: restriction, concern about food, weight concern, and concern for body shape. A global scale is obtained by summing the four subscales and dividing them by 4. The four subscales are scored, and the general scores are consistent with the presence of an eating disorder when above the z-score of 1.5. The Italian translation by Dalle Grave and Calugi was used in this study [54], showing good reliability (α = 0.89).
3. Binge-eating disorder symptomatology. The Binge-Eating Scale (BES) is a 16-item questionnaire that explores behaviors, feelings, and thoughts related to an episode of binge eating (e.g., “Sometimes I feel like I’m gulping down food. Despite this, I never end up feeling too full”). For each item, subjects can choose the statement that best represents them among the four proposed. After recoding, responses are summed together into a total score: scores below 17 indicate no or unlikely binge eating; between 17 and 27, possible or mild binge eating; and above 27, probable or severe binge eating. The scale has been translated, adapted, and validated in Italian contexts. In this study, BES showed good internal consistency (α = 0.89).
4. Eating self-efficacy. The Eating Self-Efficacy Brief Scale (ESEBS) [55] is an 8-item questionnaire that measures self-efficacy in regulating eating, defined as the extent to which one feels able to self-regulate relating to food. Answers are rated on a scale of 0 to 5, ranging from “not at all easy” to “completely easy”. The responses are summed on two factors: social self-efficacy, which measures the ability to regulate eating in social contexts (e.g., “How easy would it be for you to resist the urge to eat when eating out with friends”), and emotional self-efficacy, the ability to resist in situations of emotional activation (e.g., “How easy would it be for you to resist the urge to eat when you are worried about work/study reasons”). Both factors showed good internal consistency in this sample (social self-efficacy, α = 0.86; emotional self-efficacy, α = 0.79).
5. Psychopathological distress. The Symptom Checklist-90-Revised (SCL-90-R) [56] explores the presence of psychological symptoms related to nine areas of functioning plus an overall score. It consists of 90 questions about different symptoms (e.g., “Headache”; “Difficulty remembering things”), and the subject should indicate, on a scale from 0 (“not at all”) to 4 (“very much”), the intensity with which they have suffered from the symptom in the past week. The Italian version of the SCL-90-R [57] has been translated, adapted, and validated in a general sample of adolescents and adults. Cronbach’s alpha for this sample was 0.97.
6. Self-esteem. The Italian version of the Rosenberg Self-Esteem Scale [58,59] is a questionnaire consisting of 10 statements that measure how the subject evaluates himself (e.g., “I think I have a number of qualities”) on a 4-point scale of agreement, from 4 = strongly disagree, to 1 = strongly agree, for items 3, 5, 8, 9, and 10 and from 1 = strongly disagree, to 4 = strongly agree, for items 1, 2, 4, 6, and 7. Higher scores indicate a higher self-esteem. The questionnaire showed good internal consistency (α = 0.86).

### 2.6. Hypotheses

#### 2.6.1. Primary Outcomes

As compared to pre-treatment, at post-treatment, participants in the intervention group will show significant decreases in binge eating, eating-behavior symptomatology, and BMI, a change that is bigger than the control group.

#### 2.6.2. Secondary Outcomes

As compared to pre-treatment, at post-treatment, participants in the intervention group will show significant decreases in the indices of psychopathological distress and increases in emotional and social eating self-efficacy and self-esteem, a change that is greater than the control group. 

#### 2.6.3. Maintenance of the Post-Intervention Changes

The improvements will be maintained at a one-month follow-up.

### 2.7. Data Analyses

Data were analyzed using JASP v.0.72.2.1. Firstly, chi-square tests of independence and independent-sample t-tests were conducted to determine the baseline equivalence between the intervention and control groups on socio-demographic characteristics. Afterward, two-way mixed-design ANOVAs were implemented to examine the effects of the intervention on relevant outcomes (between-subject factor “Groups”: intervention vs. TAU; within-subject factor “Moment of the assessment”: pre- vs. post-test). Wherein ANOVAs revealed significant Group*Moment interactions, analyses of simple main effects were conducted to examine the moment effect within each group individually. Effect sizes for interactions were quantified through partial eta squared (η_p_^2^), with values of 0.01, 0.06, and 0.14 representing small, moderate, and large effects, respectively [60]. Finally, paired-sample t-tests were carried out to examine whether the post-treatment results were maintained at a one-month follow-up assessment in the intervention group (grouping variable: post-intervention vs. one-month follow-up). Effect sizes were quantified through Cohen’s d, with values of 0.2, 0.5, and 0.8 representing small, moderate, and large effects, respectively [60]. For all analyses, the significance level was set to 0.05.

## 3. Results

### 3.1. Descriptives

Sixty-five patients reporting binge-eating symptoms within subthreshold and clinical eating disorders (84.6% F; age M = 38.5 ± 13.2, BMI = 37.8 ± 9.59) were enrolled in the final sample. Participants (experimental group, N = 43; treatment-as-usual group N = 22) completed self-reported questionnaires assessing binge-eating symptoms and psychological correlates at pre-treatment (T0), post-treatment (T1), and a one-month follow-up (T2). Even though the whole experimental group completed the treatment and the questionnaires at T1 and participated in the follow-up session, only 46% of the participants (N = 20) completed the survey at T2. No significant differences were observed between the intervention group and the control group on socio-demographic characteristics, such as gender (χ^2^ [1] = 0.20, *p* = 0.655); age (t [63] = 0.691, *p* = 0.492); and BMI (t [60] = −0.995, *p* = 0.324) (see Table 1).

### 3.2. Efficacy of Web-Based Group Intervention

**Figure 1:**
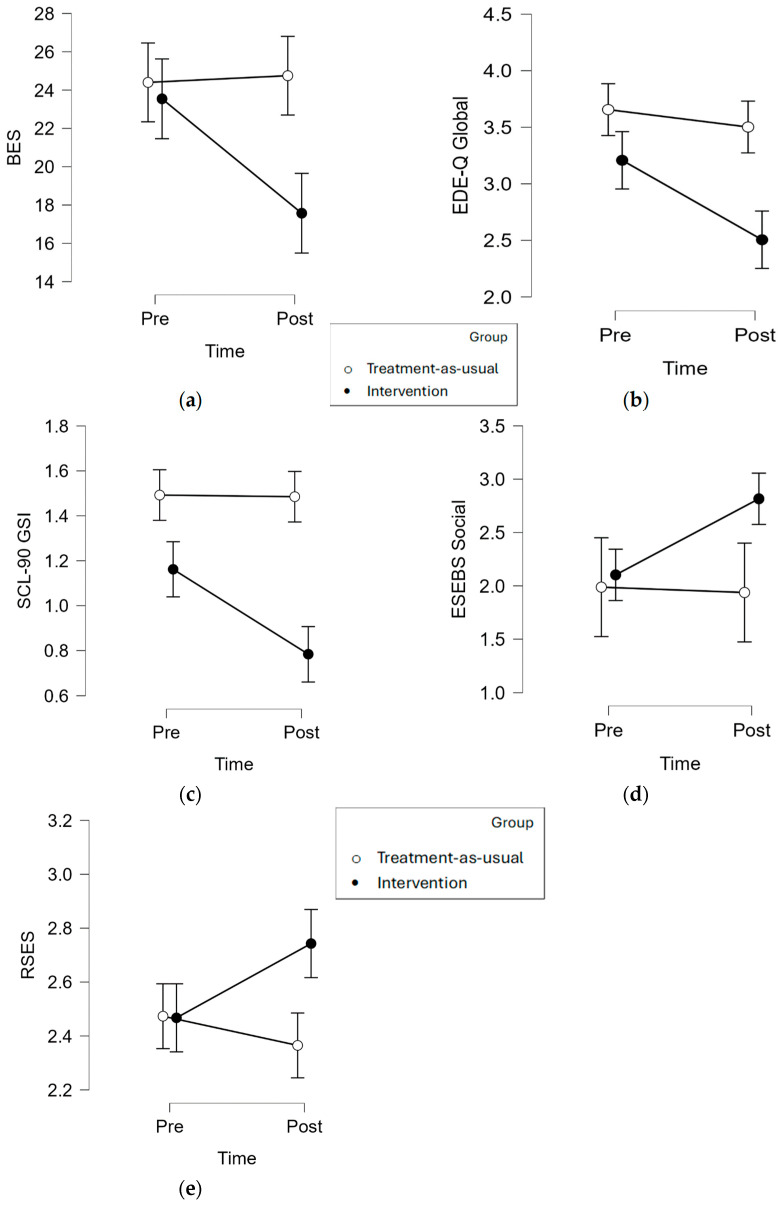
Representation of the effects of the intervention in the experimental and in the control group for the studied variables: Moment*Group interaction on (**a**) scores for Binge-Eating Scale (BES); (**b**) global scores for Eating Disorder Examination Questionnaire (EDEQ); (**c**) global severity index scores for Symptom Checklist-90-Revised (SCL-90-R); (**d**) scores for Eating Self-Efficacy Brief Scale in Social Contexts (ESEBS Social); (**e**) scores for Rosenberg’s Self Esteem Scale (RSES).

Separate two-way mixed-design ANOVAs revealed significant scores for Group*Moment interactions for binge eating (F[1, 53] = 8.357, *p* = 0.006, η_p_^2^ = 0.136); eating-behavior symptomatology (F[1, 52] = 4.487, *p* = 0.039, η_p_^2^ = 0.079); psychopathological symptoms (F[1, 54] = 8.729, *p* = 0.005, η_p_^2^ = 0.139); social self-efficacy (F[1, 52] = 5.561, *p* = 0.022, η_p_^2^ = 0.097); and self-esteem (F[1, 53] = 8.482, *p* = 0.005, η_p_^2^ = 0.138). No significant interaction was found for emotional self-efficacy (F[1, 52] = 0.889, *p* = 0.350, η_p_^2^ = 0.017), despite the mean scores going in the expected direction, and BMI (F[1, 51] = 0.028, *p* = 0.868, η_p_^2^ = 0.001). More specifically, simple effect analyses revealed that the intervention group showed significant decreases in the scores for binge eating (F[1, 53] = 17.021, *p* < 0.001); eating-behavior symptomatology (F[1, 52] = 15.895, *p* < 0.001); and psychopathology (F[1, 54] = 19.513, *p* < 0.001) as well as significant increases in the scores for social self-efficacy (F[1, 52] = 18.182, *p* < 0.001) and self-esteem (F[1, 53] = 9.814, *p* = 0.004). Conversely, no significant differences between the pre- and post-test scores were observed within the control group for binge eating (F[1, 53] = 0.064, *p* < 0.804); eating behaviors (F[1, 52] = 0.991, *p* = 0.332); psychopathology (F[1, 54] = 0.009, *p* = 0.925); social self-efficacy (F[1, 52] = 0.026, *p* = 0.875); and self-esteem (F[1, 53] = 1.775, *p* = 0.199). The results are summarized in Table 2, whilst the interaction effects are plotted in Figure 1.

### 3.3. One-Month Follow-Up for the Web-Based Group Intervention

The treatment effects on the web-based intervention group were maintained at the one-month follow-up assessment (see Table 3). Specifically, there were no significant differences between post-intervention and the one-month follow-up in the scores for social self-efficacy (t [15] = −0.207, *p* = 0.839, Cohen’s d = −0.052) and self-esteem (t [16] = 0.365, *p* = 0.720, Cohen’s d = 0.089). Notably, further decreases in the scores for binge eating (t [20] = 2.396, *p* = 0.026, Cohen’s d = 0.523); eating behavior symptomatology (t [16] = 2.336, *p* = 0.033, Cohen’s d = 0.567); and psychopathology (t [20] = 2.181, *p* = 0.041, Cohen’s d = 0.476) were observed between post-intervention and the one-month follow-up.

## 4. Discussion

The present study aimed to assess the effectiveness of a brief web-based multidisciplinary group intervention in reducing psychological distress and binge-eating-related symptomatology in a sample of patients with dysregulated eating behaviors.

The results evidence a significant reduction in binge-eating-related symptomatology and general psychopathology and an increased self-esteem and eating self-efficacy in social contexts in the treatment group compared to the TAU group (T0 vs. T1). This could be related to the effectiveness of the intervention in addressing dysregulated eating behaviors and their precursors. During the meetings, participants reported an increase in awareness and in the processes characterizing dysfunctional cycles (e.g., triggering events, vulnerability factors, repetitive patterns, judgmental thoughts, emotion identification, tolerance, and regulation). The qualitatively collected observations of the participants, especially in the last session, suggested the intervention’s acceptability and confirmed the results observed through the questionnaires (e.g., “*I’m trying not to judge myself, and I’m more aware of what I eat. I try and be mindful even when I shop at the grocery store*”; “*I was able to stop myself many times. That’s not a victory, but a beginning*”; “*Now I have many instruments*”; “*I’m understanding myself more, through inner observation, and dysfunctional eating behaviors are decreasing. I am having the clarity of choosing one thing at a time and enjoying it*”). Comments also showed the feasibility of the group setting (“*I know I will miss this group*”; “*You were like brothers within this journey*”), and positive observations were also collected about the online mode, which facilitated participation, especially considering the length of each session (2 h) and the difficulties of maintaining such a commitment, considering the daily schedule of the participants’ work day. Improvements in binge-eating symptoms, eating-behavior symptomatology, general psychopathology, and eating self-efficacy in social contexts significantly continued to rise after a one-month follow-up (T2) in the treatment group. These results cannot be considered as an implication of the prolonged effect of the intervention since one month is a short amount of time. Longer follow-up evaluations were not planned at the beginning of this study; however, such evaluations will be considered in a secondary analysis. Since the intervention is still ongoing as a part of the activities of the service, the latest and new editions of the group training will be evaluated six months and one year from the end of the intervention. No differences in the BMI and emotional eating self-efficacy at pre- and post-intervention were observed. This could be explained by the relatively small sample size and the main focus of the program on dysregulated eating behaviors, not weight reduction. Moreover, the short-term assessments may have interfered with the detection of significant differences in some of the variables that may have longer latency times than others, such as those related to weight reduction (and consequently BMI). Our main findings are in line with those of recent randomized controlled trials (RCTs) supporting the effectiveness of internet-based CBT interventions [37] and guided self-help CBT interventions [38,61] in reducing eating symptomatology in BED as well as BN [37,62]. Moreover, online DBT skills training has shown potential with different clinical populations and symptomatology (e.g., depression, suicidal ideation, heavy episodic drinking, and borderline personality disorder), as reported by a recent review [63]. All these interventions are brief (ranging from 12 to 24 weeks in length) and are based on individual completion through automatized online modules (i.e., [61]), website interactions, and telephone meetings following assignments (i.e., [38]) or a weekly internet-based asynchronous therapist contact, with very few face-to-face meetings [37]. Our intervention program benefits both the potentialities of psychologist-led therapy and group settings. This constitutes a great opportunity in terms of mirroring and sharing with others in a safe and protected environment, given the huge vulnerability of people with binge eating who feel disgusted and embarrassed and who eat alone [1]. Our sample presented a lower level of dropouts (4.65% of the treatment group) than what is reported in the literature. In fact, according to a recent review by Linardon and colleagues, the dropout rate for prevention- and treatment-focused online interventions for eating disorders is about 21%, with a high heterogeneity [35]. In the present study, the intervention and follow-up were completed by all participants, except for two, who abandoned the intervention after the first meeting, reporting that they felt that the group setting was not the right approach for them and who continued the treatment individually, consistent with the in-presence original training. On the other hand, many participants (almost half of the total) were not compliant in completing the final questionnaires after the last follow-up meeting, thus limiting our results. Motivations were not formally collected. Internet-delivered interventions are proving to be a conceivable alternative to standard treatment since treatment effects are comparable to day-patient treatment [37]. However, as suggested by two systematic reviews and meta-analyses published in 2020 and 2021 [35,36], evidence is still lacking, and fostering academic research and clinical studies in support of this affordable and sustainable resource is a priority. Despite the significant improvements in both the post-intervention and follow-up, it is relevant to acknowledge the small sample size that could affect the generalizability of the results. The present study is not an RCT since a randomized sampling was not compatible with the functioning of the public health service and the urgency of the treatment delivery, especially during and after the pandemic. Moreover, RCTs can have a limited impact on controlling confounding variables when smaller samples are implied, in contrast with realistic evaluations, which may be more reflective of the facets of individual experiences [64]. Albeit not being an RCT undermines the generalizability of the results, this study represents a first step towards promoting alternative treatment interventions that adapt to the multiplicity of factors related to disordered eating, the variety of patients, and breaking down barriers to mental health care, in particular, eating disorders. In our case, the COVID-19 pandemic and the need to respond to requests for a service compromised by isolation and the problems related to the increase in patient care requests have promoted the development and adaptation of this intervention, which proved to be effective and promising. So far, most of the published studies refer to participants recruited before the pandemic, with few exceptions. Therefore, there is an urgent need to expand and update empirical evidence on the effectiveness of validated web-based interventions with samples that truly represent the current reality of people with lived experiences of BED and BN, including the experience of the pandemic and its consequences [65].

### Strengths, Limitations, and Future Directions

To summarize, the results of our study should be interpreted cautiously, considering (a) the non-randomized allocation of the participants into groups, which could be representative of the reality of clinical settings; (b) the small sample size, particularly concerning the follow-up analysis, which may result in underpowered tests; (c) the lack of structured acceptability and feasibility measures; (d) the lack of adherence of the participants who did not appreciate the group setting; and (e) the short-term follow-up and the scarce compliance in completing all its measures. Despite these limitations, the results support the effectiveness of a brief web-based multidisciplinary group intervention in reducing eating symptomatology and psychological distress and enhancing self-esteem and eating self-efficacy in a group of people with dysregulated eating behaviors. This study also has some strengths, namely, (a) its *in-field* nature being an evaluation of the real functioning of the service; (b) the positive feedback of participants, as registered during the meetings; (c) the affordability and sustainability related to the online modality, which facilitates the participation of both clinicians and participants; (d) the use of evidence-based techniques (i.e., the DBT for BED and BN skills training) together with multimedia (such as video clips) that is effective and captivating; (e) the multidisciplinary implementation of the training, which also contains a session of therapeutical education about one’s diet and obesity management; and (f) the involvement of various psychologists (i.e., 10), which could decrease the risk of subjective biases in the intervention delivery, who were supervised by experts in the field, to ensure an adequate and clinically effective administration.

Further research should confirm the results with a larger sample and within an RCT methodology. Moreover, future studies should explore more facets of the intervention’s effectiveness. For example, evidence should be collected about the effectiveness of the individual administration experiences that have been conducted in cases where the group setting is not accepted or incompatible. Finally, further research is needed to evaluate the state of the improvements after six months or one year from the intervention.

## 5. Conclusions

This study described the effectiveness of a brief multidisciplinary web-based group intervention for 65 Italian patients with clinical and subthreshold bulimia and binge-eating disorder in a three-year experience within a multidisciplinary public clinical setting. The intervention demonstrated good potential, improving eating behaviors, decreasing psychological symptoms, and increasing eating self-efficacy and self-esteem, with stability for one month. This research contributes evidence to the ongoing study of affordable, accessible treatments for disordered eating behaviors, which is in its early stages. Further studies should explore the maintenance of the results with a longer follow-up time.

## Figures and Tables

**Table 1:** Socio-demographic characteristics of the sample.

	Total Sample	Intervention	TAU	*t*-Test/χ² Test (df)	Effect Size
	Mean (SD)/*n*	Mean (SD)/*n*	Mean (SD)/*n*		
**Gender**	10 m; 55 f	6 m; 37 f	4 m; 18 f	0.20 (1)	Phi coefficient = 0.055
**Age**	38.51 (13.15)	37.7 (13.05)	40.09 (13.51)	0.691 (63)	Cohen’s d = 0.181
**BMI**	37.85 (9.59)	38.75 (10.43)	36.21 (7.81)	−0.995 (60)	Cohen’s d = −0.264

Abbreviation: df, degree of freedom; SD, standard deviation; m, male; f, female; BMI, Body Mass Index.

**Table 2:** Efficacy of web-based group intervention (estimated marginal means from two-way mixed ANOVAs).

	Intervention (*n* = 43)	TAU (*n* = 22)	Moment × Group Interaction		
Variable	Mean (SE)	Mean (SE)	F Value	*p*	η_p_^2^
**BMI**			0.028 [1, 51]	0.868	0.001
Pre	39.4 (1.7)	36.9 (2.2)			
Post	38.8 (1.8)	36.3 (2.3)			
**BES**			8.357 [1, 53]	**0.006**	0.136
Pre	23.5 (1.7)	24.4 (2.2)			
Post	17.6 (1.7)	24.7 (2.3)			
**SCL-90-R GSI**			8.729 [1, 54]	**0.005**	0.139
Pre	1.16 (0.10)	1.49 (0.13)			
Post	0.78 (0.08)	1.49 (0.10)			
**EDEQ Global**			4.487 [1, 52]	**0.039**	0.079
Pre	3.20 (0.21)	3.66 (0.28)			
Post	2.50 (0.20)	3.50 (0.25)			
**ESEBS Social**			5.561 [1, 52]	**0.022**	0.097
Pre	2.10 (0.25)	1.99 (0.32)			
Post	2.82 (0.25)	1.94 (0.32)			
**ESEBS Emotional**			0.889 [1, 52]	0.350	0.017
Pre	1.01 (0.19)	1.01 (0.25)			
Post	1.82 (0.22)	1.46 (0.29)			
**RSES**			8.482 [1, 53]	**0.005**	0.138
Pre	2.47 (0.09)	2.47 (0.12)			
Post	2.74 (0.091)	2.36 (0.12)			

Abbreviations: SE, standard error; BMI, Body Mass Index; BES, Binge-Eating Scale; SCL-90-R GSI, Symptom Checklist-90-Revised Global Severity Index; ESEBS, Eating Self-Efficacy Brief Scale; RSES, Rosenberg’s Self-Esteem Scale. Bold indicates significative differences.

**Table 3:** One-month follow-up for the Web-Based Group Intervention (Paired samples t-tests between post-intervention and one-month follow-up).

	Post-Intervention	One-Month Follow-Up		
	Mean (SD)	Mean (SD)	t Value (df)	Cohen’s d
**BES**	19.286 (10.311)	17.333 (10.389)	2.396 * (20)	0.523
**EDEQ Global**	2.700 (1.206)	2.423 (1.055)	2.336 * (16)	0.567
**SCL-90-R GSI**	0.804 (0.444)	0.732 (0.425)	2.181 * (20)	0.476
**ESEBS Social**	2.594 (1.147)	2.656 (1.161)	−0.207 (15)	−0.052
**RSES**	2.618 (0.631)	2.582 (0.547)	0.365 (16)	0.089

* *p* < 0.05. Abbreviations: BES, Binge-Eating Scale; EDEQ, Eating Disorder Examination Questionnaire; SCL-90 GSI, Symptom Checklist-90 Global Severity Index; ESEBS, Eating Self-Efficacy Brief Scale; RSES, Rosenberg’s Self-Esteem Scale; SD, standard deviation; df, degree of freedom.

## Data Availability

The data presented in this study are available on request from the corresponding author. The data are not publicly available due to privacy and ethical restrictions.

## Supplementary Materials

The following supporting information can be downloaded at: https://www.mdpi.com/article/10.3390/nu16162696/s1, Intervention description: the intervention.
