# Supplementary material for: A Brief Online Intervention Based on Dialectical Behavior Therapy for a Reduction in Binge-Eating Symptoms and Eating Pathology
Source: Nutrients. 2024 Aug 14;16(16):2696. doi: 10.3390/nu16162696 (PMC11357140; doi:10.3390/nu16162696)
Supplement: Supplementary file 1 [file nutrients-16-02696-s001.zip › nutrients-3110243-supplementary.pdf]

Supplementary Material

Intervention

Theoretical Framework

Dialectical Behavior Therapy (DBT) protocols aim at reducing behaviors that interfere with psychological health and everyday activities, decreasing one's quality of life. The dialectical behavior model explains binge eating and purging behaviors as a dysfunctional emotional regulation strategy within a chain of variables triggered by an internal or external event. Those dysfunctional behaviors serve as a coping mechanism to decrease negative emotions activated by the event in the short term, thus contributing to the maintenance of the disorder. In the medium and long term, however, this mechanism leads to negative consequences such as low self-esteem, guilt and shame, less functional emotional regulation, and costs for physical health. The protocol consists of training in three of the four skills initially conceived by Linehan, namely mindfulness, emotion regulation, and distress tolerance. The Skills are explored within modules: Mindfulness involves the development of some core abilities, such as observing, describing, participating spontaneously at the moment, and being non-judgmental toward themselves. Such abilities are especially contextualized into eating behaviors, for example, eating with awareness, and enjoying the meal. Emotion regulation focuses on a better comprehension of emotions and a greater self-emotional awareness and also provides alternative regulatory strategies to food. Finally, stress tolerance addresses the capacity to accept stressful situations and overcome crises, taking distance from dysfunctional situations and taking the time to examine the pros and cons.

Structure and content of each session

Consistently with the DBT approach, the first meeting includes the pre-treatment stage with the treatment agreement and the establishment of the therapeutic alliance, as well as psychoeducation over the DBT model of dysregulated eating behavior. The following meetings included the discussion of the homework and psychoeducation and exercises, on the three Skills of DBT included in the original protocol, namely mindfulness, emotion regulation, and distress tolerance. The ninth meeting included a therapeutical education with a Physician Specialist in Nutritional Science, focused on themes such as obesity management, false myths about food and nutrition, or information about fad diets. The last meeting occurred one month after the ninth with the objective of consolidating the new skills and monitoring. The intervention included multimedia as a tool to increase comprehension of the theme. The main topics of each meeting are summarized in Table S1.

Table S1. Session content of each of the web-based 10 sessions.

session	skill	main topics
1	Pre-treatment	Treatment agreement; introducing the group; chain analysis
2	Mindfulness	Rational mind, affective mind, wise mind
3	Mindfulness	observe, describe, participate
4	Mindfulness	not judgmentally; one-mindfully; effectively
5	Emotion Regulation	observing and describing emotions and their function
6	Emotion Regulation	Increasing positive emotions and experiences; regulation strategies
7	Distress Tolerance	Skills to accept reality (e.g. observing the breath)
8	Distress Tolerance	Crisis survival skills (e.g. pros and cons)
9	Nutrition Knowledge	Therapeutic education on nutrition, diets, and obesity management
10	One-month Follow-up	Relapse prevention

Missed sessions

In order to increase adherence to the intervention and guarantee 100% participation for every participant, one-hour recovery sessions were organized for each participant who couldn't attend one to two meetings. This was organized in accordance with the original authors' recommendations and the commitment agreement signed after the first meeting. Patients benefited from this opportunity with compliance, informing in advance of their absence or being late.
